# Potential Targets to Mitigate Trauma- or Sepsis-Induced Immune Suppression

**DOI:** 10.3389/fimmu.2021.622601

**Published:** 2021-02-25

**Authors:** Christian B. Bergmann, Nadine Beckmann, Christen E. Salyer, Marc Hanschen, Peter A. Crisologo, Charles C. Caldwell

**Affiliations:** ^1^ Division of Research, Department of Surgery, College of Medicine, University of Cincinnati, Cincinnati, OH, United States; ^2^ Experimental Trauma Surgery, Klinikum rechts der Isar, Technical University of Munich, Munich, Germany; ^3^ Department of Trauma Surgery, Klinikum rechts der Isar, Technical University of Munich, Munich, Germany; ^4^ Division of Podiatric Medicine and Surgery, Critical Care, and Acute Care Surgery, Department of Surgery, College of Medicine, University of Cincinnati, Cincinnati, OH, United States; ^5^ Division of Research, Shriners Hospital for Children, Cincinnati, OH, United States

**Keywords:** IL-10, transforming growth factor β, thymic stromal lymphopoietin, immunosuppression, chronic critical illness

## Abstract

In sepsis and trauma, pathogens and injured tissue provoke a systemic inflammatory reaction which can lead to overwhelming inflammation. Concurrent with the innate hyperinflammatory response is adaptive immune suppression that can become chronic. A current key issue today is that patients who undergo intensive medical care after sepsis or trauma have a high mortality rate after being discharged. This high mortality is thought to be associated with persistent immunosuppression. Knowledge about the pathophysiology leading to this state remains fragmented. Immunosuppressive cytokines play an essential role in mediating and upholding immunosuppression in these patients. Specifically, the cytokines Interleukin-10 (IL-10), Transforming Growth Factor-β (TGF-β) and Thymic stromal lymphopoietin (TSLP) are reported to have potent immunosuppressive capacities. Here, we review their ability to suppress inflammation, their dynamics in sepsis and trauma and what drives the pathologic release of these cytokines. They do exert paradoxical effects under certain conditions, which makes it necessary to evaluate their functions in the context of dynamic changes post-sepsis and trauma. Several drugs modulating their functions are currently in clinical trials in the treatment of other pathologies. We provide an overview of the current literature on the effects of IL-10, TGF-β and TSLP in sepsis and trauma and suggest therapeutic approaches for their modulation.

## Immune Perturbation in Sepsis and Trauma

Infection and injury can lead to an increase of alarm signals that signal the presence of invading pathogens or injured tissue. The so called `danger hypothesis` was developed by Matzinger et al. stating that alarmins are either Pathogen-Associated Molecular Patterns (PAMPs) or Damage-Associated Molecular Patterns (DAMPs) (1). Either may trigger inflammation by recognition through Pattern Recognition Receptors (PPRs) on innate and adaptive immune cells (2). Within these cells, the nuclear translocation of the transcription factor Nuclear Factor kappa beta (NF-κB) promotes pro-inflammatory cytokine production to include IL-1 and TNF-α (3). These cytokines act upon a variety of target cells (4) that can begin a cascade of increasing pro-inflammatory cytokines, such as IL-6, IL-8, and MIF (5). Increased production of these cytokines can result in the induction of fever (6), the upregulation of adhesion receptors like ICAM-1 on endothelial cells (7), increased L-selectin (CD62L) on lymphocyte cells (8) and neutrophil mobilization. Excessive inflammation can lead to excessive damage tissue and organs, e.g. by the release of radicals by neutrophils (9) to include the lungs, heart, kidney, liver and brain (10). In addition, disseminated intravascular coagulation (DIC) and thrombocytopenia (11) can occur, which are known to contribute to multiple organ dysfunction syndrome (MODS) (12). This cytokine driven initial inflammation can lead to early death if not compensated (13).

Concurrent with the pro-inflammatory response, there is evidence of a number of immune suppressive events occurring. These include the loss of conventional T lymphocytes (14) simultaneous with the lack of regulatory T cell apoptosis (15), the increased number of myeloid suppressor cells (16) coupled with the down regulation of monocyte HLA-Dr (17), and a loss of dendritic cells (18). Additionally, there are increases in immune suppressive cytokines such as IL-10, TGF-ß, and TSLP. Significant immune suppression can impair the proper clearance of pathogens leading to higher bacterial loads both systemically and in tissues. According to a recent clinical observational cohort study, septic patients can develop chronic critical illness, with a 6-month survival of 63%, and continue to demonstrate cytokine profiles of chronic inflammation, as well as biomarker profiles characteristic for persistent immunosuppression (19). Additionally, these patients very often require treatment in long term acute care facilities, and by one year after hospital discharge half of them have died and another quarter remains bedridden (20). Elder patients are more likely to develop these immune disbalances (21). It is therefore key to better understand these pathophysiologies as they become an increasing problem for aging societies (22). It is beyond the scope of this review to detail the entire host response to sepsis and this topic is covered in a number of other reviews (23–25). Here, this review focuses upon release, impact, and potential therapy of a limited number of potent anti-inflammatory cytokines.

## Mediators of Immunosuppression

The initial upregulation of inflammation after onset of infection or injury results in the activation of the innate immune system, mostly of neutrophils and macrophages *via* Toll like receptors (TLRs) (26, 27). Orchestrated by T cells, they maintain inflammation by the release of pro-inflammatory cytokines such as TNF-α, IL-6, IL-8, MCP-1, MIP-1α (28, 29), among which IL-6 plays a prominent role as it is associated with injury severity (30), MODS and death (31). At the same time, immunosuppression is induced (13), which is strongly driven by anti-inflammatory cytokine release (32). Immunosuppression is characterized by the induction of immunosuppressive functions and partial impairment of pro-inflammatory functions of innate and adaptive immune cells: lymphopenia occurs as the number of circulating lymphocytes decreases due to apoptosis of CD4 and CD8 T cells, B cells, and NK cells (33), Regulatory T cells numbers increase after sepsis or trauma (34, 35), T cells become exhausted and anergic (36, 37), monocytes, macrophages and dendritic cells become impaired in their ability to mount a proinflammatory response (22, 38, 39), neutrophil dysfunction ensues (40) and increased release and activation of immunosuppressive Myeloid Derived Suppressor Cells (MDSCs) is triggered (41). These cells, along with other tissue cells, such as endothelial cells, potently mediate immunosuppression by the release of anti-inflammatory cytokines. The cytokines IL-10, TGF-β and TSLP all exert potent anti-inflammatory functions (42–44). In trauma and sepsis, IL-10 is considered a key immunosuppressive cytokine and correlates with injury severity and outcomes (45) and is widely examined in sepsis and trauma studies. However, it has been demonstrated that all three cytokines also exert pro-inflammatory functions under certain conditions (46–48). This seems contradictory as they are considered key regulators of immunity. In the following chapters, we will review their immunosuppressive mechanisms and dynamics in sepsis and trauma. Finally, the pathological conditions in sepsis and trauma that may drive a detrimental release of these cytokines will be examined.

## Immunosuppressive Cytokines: Il-10, TGF-β, and TSLP

### IL-10

#### How Does IL-10 Affect Immune Cells?

With its potent anti-inflammatory functions IL-10 is considered an important regulatory cytokine protecting the host from exaggerated inflammation, autoimmunity and allergy (49, 50). It is a V-shaped homodimer and has a molecular weight of 37 kDa (51) and it is a key member of the IL-10 superfamily which consists of IL-19, IL-20, IL-22, IL-24, IL-26 and the type III IFN-γ subfamily (51).

In sepsis and trauma, IL-10 mainly affects immune cells rather than tissue cells, which do not express the IL-10-receptor (IL-10R) to the same extent. Tissue cells are more affected by other member of the IL-10 super family, particularly IL-22 (50). Many variants of the IL-10 gene exist, and an increasing body of literature suggests that IL-10 gene polymorphisms play an important role in different pathological conditions (52). Tumor cells can also produce IL-10 and it is thought that this allows the tumor to mitigate an effective immune response (51).

The IL-10 receptor is a composition of the IL-10R2 and IL-10R1 subunits, with the latter being expressed mainly on immune cells. The expression of the receptor differs from cell type to cell type. For example, some monocytes and macrophages express very high amounts of the receptor, and the expression in memory T cells is higher than in naïve CD4 T cells (53). The IL-10 cytokine binds to IL-10R1, which subsequently forms a complex with IL-10R2. It is of note that IL-10 can bind to IL-10R1 alone but not to IL-10R2. However, the affinity for the complex is much higher than for IL-10R1 alone (54). The IL-10 receptor complex then phosphorylates STAT3 *via* Jak1 and Tyk2 activation but also STAT1 and STAT5. Among these, STAT3 seems to be most important for modulating the downstream transcription of target genes (42, 51, 54). It is of importance that STAT3 also acts downstream from IL-6, so the resulting modulation depends on A) the cell type, B) the competing stimuli the cell receives and C) the duration of the stimuli (42, 51, 54). Inflammation is potently suppressed by IL-10 through inhibition of NF-κB DNA binding activity (53). IL-10 also hinders TLR signaling through induction of inhibitory miRNAs targeting MyD88-dependent TLR4 signaling and through promoting the ubiquitin-mediated degradation of IRAK1/4 and TRAF6. MyD88-independent attenuation was also reported (55). On a translational level, IL-10 leads to an increase of tristetrapoline, a protein which downregulates mRNA of pro-inflammatory cytokines and can be down-regulated itself in TLR-dependent pathways (55). However, IL-10R binding also limits its own effect by inducing suppressor of cytokine signaling (SOCS)3, which forms a negative feedback loop by inhibiting, amongst others, STAT3 phosphorylation (56). IL-10 also suppresses the production of pro-inflammatory cytokines TNF-α, IL-1, IL-5, IL-8, IL-12, GM-CSF, MIP-1α and MIP-2α in monocytes, macrophages, neutrophils and NK cells (57).

A wide range of immune cells can produce IL-10, such as dendritic cells (DCs), macrophages, mast cells, natural killer cells (NK), eosinophils, neutrophils, B cells, CD8 T cells, type 1 T-helper cells (Th1), Th2, and Th17 CD4 T cells, regulatory T cells, TCRαβ^+^ double negative (CD4^-^ CD8^-^) T cells (DN T cells), and myeloid derived suppressor cells (MDSCs) (49, 50, 58).

The effect of IL-10 on innate and adaptive immune cells differs. Macrophages and monocytes are suppressed by IL-10 through the down regulation of MHC II and costimulatory molecule expression (CD80, CD86, and CD40), both constitutively and in response to IFN-γ (49, 51, 59, 60). This prevents macrophages and monocytes from presenting their respective antigens to Th1 T cells, which in turn do not become activated to orchestrate an immune response. Additionally, the NO production of macrophages is reduced, adhesion to endothelial cells is hindered and the immunosuppressive M2-type is induced (51, 54). Interestingly, IL-10 promotes macrophage phagocytosis of cell debris (54).

Unlike macrophages, neutrophils need to be primed in order to become responsive to IL-10. Neutrophils express IL-10R2 constitutively but can only form the IL-10R complex when IL-10R1 gets upregulated after stimulation. Experimentally, this was determined to take about 4 h (61). Therefore, an immediate response to IL-10 is not possible in non-primed circulating neutrophils. Another important effect that can be seen upon local administration of IL-10 in inflammatory models is that IL-10 prevents the recruitment of neutrophils to the site of injury. Neutrophil migration was not directly hindered by IL-10, but the production of chemoattractants by macrophages or the affected organ tissue was decreased (61, 62). The inhibition of neutrophil apoptosis has been shown to be counter-regulated by IL-10 in severe sepsis (63). Further, it has been demonstrated that IL-10 can inhibit the production of TNF-α and IL-1β in neutrophils when stimulated with LPS or by the phagocytosis of bacteria (64). Further, ROS production seems to be diminished by IL-10 (54). However, further experiments are needed to systematically elucidate the impact of IL-10 on neutrophil functionality. Interestingly, under certain conditions IL-10 induces its own production in neutrophils and macrophages, which is thought to be a positive feedback loop in innate immunosuppression (65). This is of interest as neutrophils themselves are contributing significantly to IL-10 production as shown in a murine model of abdominal sepsis (66).

In dendritic cells, MHC II expression and therefore antigen presentation and activation of naïve CD4 T cells is impaired by IL-10 (54). This potently inhibits CD4 T cell functions. It also diminishes the production of T cell pro-inflammatory cytokines, such as IL-2 and IFN-γ (51). Further, IL-10 blocks proliferation of Th17 cells and inhibits Th2 cells (42, 67). It also promotes the proliferation and function of Regulatory T cell subsets. The generation of Type-1 regulatory T cells (Tr1 T cells) from naïve T cells is induced by IL-10 and it promotes survival and function of CD4^+^ FoxP3^+^ Tregs (49, 67). For example, Treg expression of FoxP3 appears to be IL-10 dependent (68). Interestingly, IL-10 suppresses thymic lymphocyte apoptosis in sepsis (69) and has stimulatory effects on CD8 T cells. When stimulated with IL-4 and/or IL-2, CD8 T cells showed increased proliferation (51). Recent murine studies showed increased IFN-γ production by CD8 T cells upon IL-10 administration (53, 70). In clinical trials it could even be shown that modified IL-10 reduces tumor burden in renal cell cancer (71). The application of modified IL-10 enhanced intratumoral CD8 T cell expression of IFN-γ and Granzyme B (42, 71) and thereby promoted anti-tumor cytolysis. However, a final conclusion cannot be drawn yet as IL-10 does also have tumor-supportive functions, i.e., enabling tumor cells to escape an effective immune response (51).

Another pro-inflammatory function of IL-10 can be seen in B cells, as it promotes their survival, proliferation and differentiation into plasma cells (51, 53). Conflicting data exists about the effect of IL-10 on NK cells. It has been shown that IL-10 suppresses TNF-α and IFN-γ production by NK cells, but promotion of IFN-γ production has also been reported in *in vivo* and *in vitro* models (51, 53, 72). Most likely, the predominant conditions, timing and dose in the respective organ or tissue lead to increased or diminished functionality.

**Figure 1:**
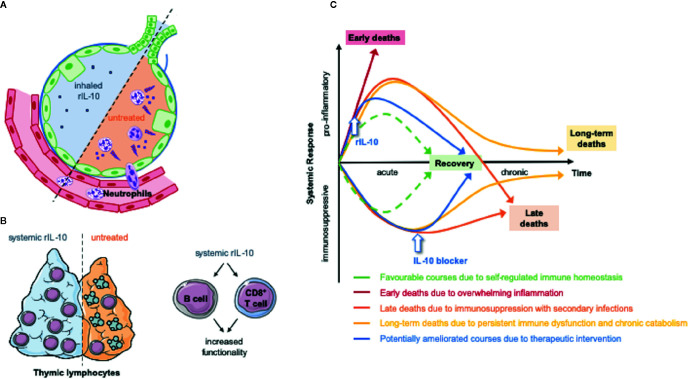
The effect of therapeutic IL-10 modulation depends on the mode of administration and dynamics. The systemic or local application of recombinant IL-10 have experimentally shown beneficial effects. **(A)** Inhaled IL-10 attenuated pulmonary inflammation by preventing detrimental neutrophil recruitment into the lung (61, 62, 73). **(B)** Moreover, IL-10 suppresses thymic lymphocyte apoptosis in sepsis (69) and has stimulatory effects on CD8 T cells (53, 70) and B cells (51, 53). Both modes of application might represent future therapeutic approaches in humans. **(C)** The dynamics of the immune perturbation in trauma and sepsis was modeled in recent literature (74). In general a time dependent modulation of IL-10 levels might restore immune hemostasis (75–77). Animal models suggest a beneficial effect of early IL-10 application (76) and the correlation of elevated IL-10 levels and injury severity suggests beneficial effects by IL-10-blockage in later phases (31, 45, 78). The figure contains adapted graphics from Les Laboratoirs Servier - Medical Art under the terms of the Creative Commons Attribution License (CC BY) for non-commercial use. The use, distribution or reproduction in other forums is permitted: https://creativecommons.org/licenses/by/3.0/legalcode, last accessed July 30th, 2020.

Different effects following systemic or local administration of IL-10 could also be utilized therapeutically. Two murine trauma studies showed that IL-10 locally administered *via* inhalation in systemic inflammation attenuated pulmonary inflammation with little or no systemic side effects (62, 73). Altogether, additional research will be needed to elucidate the seemingly paradoxical findings regarding the effect of IL-10 on multiple types of immune cells. The effects of IL-10 depending on the mode of administration are highlighted in **Figure 1**.

#### Dynamics in Trauma and Sepsis

The immune perturbation in trauma and sepsis is characterized by the dichotomy of a potent pro-inflammatory upregulation of immunity and immunosuppression (13). During immunosuppression, anti-inflammatory cytokines including IL-10 are released. In search for a marker predicting the severity and outcome of the systemic inflammation, IL-10 has been studied in many animal and clinical studies. There are three broad categories that have been investigated: 1) traumatic injury (mostly burn) 2) sepsis and 3) major surgery, which all lead to a systemic inflammation.

Although studies examining trauma and sepsis do not examine the same clinical conditions leading to systemic inflammation, they share many similarities in the expression of IL-10. Some studies did not show significant differences in IL-10 serum expression between healthy controls, septic patients and patients with septic shock (75, 79). However, the major body of clinical studies examining sepsis does demonstrate this phenomenon.

Huang et al. quantified IL-10 levels in 106 burn patients and showed increased IL-10 levels in septic vs non-septic patients (45). In another study Stensballe et al. examined 265 trauma patients. They found a significant increase of IL-10 levels in patients not surviving 30 days and a positive correlation of IL-10 levels and the injury severity score (ISS) (78). A further multicenter study including 54 polytrauma patients found that elevated IL-10 levels and an elevated IL-6/IL-10 ratio were directly proportional with MODS and mortality (31). In a clinical trial, Chen et al. studied 37 thermally injured patients with a total burn surface area >20% and found increased IL-10 levels in patients who developed sepsis versus those that did not (80).

The team around Gogos et al. included 65 patients with severe sepsis in their trial and found a positive association between serum IL-10 levels, and a high IL-10 to TNF-α ratio and death (81). In another large clinical study of 153 patients with severe sepsis and septic shock, the levels of IL-10 were significantly higher in patients that died within 48h but not for patients that died between 48h and 28 days or after 28 days (82). A separate clinical study by Frencken et al. included 708 patients with severe sepsis or septic shock in the ICU and demonstrated that higher IL-10 levels were positively associated with mortality (83). Another sizable study from Van Vught et al. in 2016 found overexpression of IL-10 signaling genes in septic patients admitted to the ICU. They did not find differences in the gene expression of IL-10 signaling between patients who later developed ICU-acquired infection compared to those who did not (84). Serum IL-10 levels were not measured, and translational and post-translational regulation of IL-10 expression was observed (85). From these results we extrapolate that sepsis appears to increase IL-10 production and signaling. Whether this indicates adverse outcomes in terms of secondary infection in septic patients has to be examined by assessment of their serum levels.

In cases of sterile trauma associated with major surgery, 28 out of 40 studies found increased systemic IL-10 levels in patients (28). An additional review by Easton and Balogh found a similar conclusion (86).

Together, these studies overwhelmingly suggest that detrimental outcome and elevated IL-10 production are associated. Further, some studies provide interesting additional findings. First, IL-10 levels correlate with the severity of the injury: elevated IL-10 serum levels correlated with the ISS of trauma patients (78), were directly proportional with MODS and mortality in a trauma population (31), showed linear correlation with the increasing percentage of burned total body surface area (45) and the IL-10/lymphocyte ratio was even correlated with the APACHE II score in severe septic patients (87). Secondly, the increase in IL-10 can be seen almost immediately after the onset of trauma, sepsis or surgery. Blood draws of the aforementioned studies were conducted either at admission to the ER or ICU (78, 83) or within 24 h after onset of the trauma, disease or surgery (45, 82, 87). This is of clinical relevance if IL-10 is used to assess the immunological disturbance in a patient at admittance in the ER or the ICU.

#### Effects and Drivers of Pathological Release in Trauma and Sepsis

The regulatory anti-inflammatory functions are considered a protective counter-regulation to prevent excessive inflammation in the balance of immune hemostasis. However, IL-10 does make the host more susceptible to overwhelming infection if it impairs a proper immune response, and thus has been associated with worse outcomes in infection and cancer (54, 88). It is currently unclear when and how supportive stimulating functions and protective anti-inflammatory regulation deteriorate under pathologic conditions. Therefore, three major questions should be addressed in future studies. A) Which stimuli drive IL-10 expression and how are these signals transmitted in the cells? B) What are the dynamics of IL-10 expression and when does IL-10 become detrimental to an orchestrated clearance of bacteria and cell debris? C) Which cell type drives IL-10 expression in which diseases?

It is known that DAMPs and PAMPs induce IL-10 production by a variety of receptors, that different bacterial pathogens provoke different expression patterns and that IL-10 regulating transcription factors differ in different T cells subsets, macrophages and neutrophils (54). In addition, IL-10 expression is also regulated post-transcriptionally (51). In myeloid cells, TLR-induction, including 2, 4, 5, 7 and 9, drives IL-10 production (54, 89). It is important to differentiate between macrophages and neutrophils in the downstream signaling as they differ. A study conducted by Tamassia et al. found that the MyD88-independent pathway is not mobilized after TLR4 stimulation in human neutrophils (90).

Further, the understanding the dynamics of IL-10 expression is essential. Several studies showed time-dependent effects of IL-10 administration or inhibition in sepsis models (75–77). Given the fact that IL-10 expression is upregulated quickly after trauma and sepsis, as described in the previous section, it seems likely that in the beginning, lower levels of IL-10 may stimulate bacterial clearance but impair functions when the serum levels exceed a certain threshold. A murine study showed increased survival in septic mice when treated with IL-10 up to 6h after the onset of sepsis, which extended the therapeutic window to conduct surgical infectious site control until 30 h after the commencement of sepsis (76).

CD4+FoxP3+Tregs are considered an important source of IL-10 in certain sepsis models (45). It is likely that the dynamic of the apoptosis of T cells in general, and the corresponding proportional changes in the T cell population affect proper IL-10 production and distribution of IL-10 (91). It is important to know which cell type contributes the most to IL-10 production in different diseases. As CD4+FoxP3+Tregs reveal an increase in frequency after trauma it is likely that they significantly contribute to IL-10 production (45). However, T cell apoptosis and murine sepsis models question this assumption. In murine abdominal sepsis, neutrophils are significant producers of IL-10 (66, 92). Of note, in murine burn models, a significant number of IL-10 producing neutrophils were found in the spleen post-injury (54). It has been shown that not only murine- but also human neutrophils can produce IL-10 (65).

Besides CD4+FoxP3+Tregs and neutrophils, also macrophages can produce IL-10 (93) and TGF-β (94) after phagocyting apoptotic antigen presenting cells (APC’s), a process coined efferocytosis (95). In this process APC’s, such as neutrophils clear bacteria *via* phagocytosis, become apoptotic and then are ingested by macrophages (96). This serves to resolve the inflammation, tissue repair and restores immune homeostasis (94, 97). Efferocytosis has been shown be impaired in sepsis, and if promoted seems to improve outcome in sepsis (98, 99) and trauma (100). In some (98, 101) but not all (99) sepsis and infection models an increase of IL-10 was shown. What remains to elucidate is whether macrophage efferocytosis contributes to chronic immunosuppression by the release of IL-10 and TGF-β. To our knowledge no studies have yet been conducted examining their role in long term (>7 days) immunosuppression after trauma or sepsis.

To assess which cell type contributes the most to IL-10 production is technically difficult. However, this should be addressed when assessing IL-10 mediated effects in the respective pathological condition, as it is necessary for evaluating any potential therapeutic approaches. Modulation of IL-10 has shown promising results in several clinical trials on autoimmune diseases (42). However, the chronic nature of these diseases and the steadier production of IL-10 varies from the highly dynamic and heterogenous pathology in systemic inflammation due to sepsis or trauma. This may provide some understanding on why past attempts to modulate IL-10 in murine sepsis models did provide conflicting results regarding their outcome (69, 102, 103). Human interventional experimental endotoxemia trials (104, 105) and *in vitro* functional assessment of the innate and adaptive immune system of sepsis patients (46), both modulating IL-10 levels, could not yet provide a clear rationale for IL-10 modulation in a specific inflammatory disease that would allow clinical trials modulating IL-10 production in sepsis and trauma. A better understanding of the dynamics and the producing cell types would significantly improve diagnostics and therapeutic advances.

#### Potential Therapeutic Modulation of IL-10

Regarding the dynamics and functionality of IL-10, we conclude that two therapeutic modulations of IL-10 in trauma and sepsis seem promising: First, inhalation of IL-10 to treat Acute Respiratory Distress Syndrome (ARDS). This pathology occurs from pulmonary infection or systemic insults such as systemic inflammation in trauma and sepsis (106). Neutrophil infiltration potently contributes to pulmonary inflammation in ARDS and leads to significant pulmonary damage (106). In two different murine trauma models inhaled IL-10 attenuated pulmonary inflammation without systemic immunosuppression by preventing the recruitment of neutrophils to the site of injury through reducing the production of chemoattractants by macrophages or the affected organ tissue (61, 62, 73)

Secondly, systemic administration of recombinant IL-10 for the treatment of sepsis can be considered. However, this approach must be carefully executed as the systemic effects are not yet fully understood. Animal studies show that the effects of IL-10 modulation depend on the injury dynamic in sepsis (75–77, 102). Murine abdominal sepsis studies suggest that the administration of IL-10 within hours after septic onset may have beneficial effects (76, 102). An endotoxemia model in primates seem to support these findings, as early intravenous injection of recombinant human IL-10 significantly decreased TNF-α, IL-6, IL-8 and IL-12 release (107). If this results in a better outcome still has to be examined in further studies, as another murine abdominal sepsis model did not find a beneficial effect of IL-10 administration on mortality (108). In the acute phase IL-10 is suggested to promote protective immunosuppressive effects, whereas in later phases of sepsis increased IL-10 is depicted as a marker for detrimental outcome in many human studies (45, 78, 81, 83). Blockage of IL-10 production or function might constitute a therapeutic approach at this point, as some animal models suggest (102). Pharmaceutic drugs for IL-10 modulation are already in clinical trials. An overview of current IL-10 modulating drugs is provided in **Table 1**. These might be considered to use to augment IL-10 function in very acute sepsis phases (Pegilodecakin: PEGylated recombinant human IL-10 (LY3500518 or AM0010); Phase 3 NCT02923921: pancreatic cancer) or to impair its function in later phases (AS101: tellurium based small compound with immune-modulating characteristics attributed to direct inhibition of IL-10; Phase 2 NCT00926354: treatment of thrombo-cytopenia in solid tumor patients). How a potential dynamic modulation of IL-10 might look like is outlined in **Figure 1**.

**Table 1:** Clinical Trials targeting IL-10.

Cytokine	Drug	Sponsor	Description	Indication	Clinical trial
**IL-10**	Prevascar	Renovo	Recombinant human IL-10	Wound healing	Phase 2 NCT00984646
	AS101	BioMAS Ltd	Tellurium based small compound with immune-modulating characteristics attributed to direct inhibition of IL-10	Treatment of thrombo-cytopenia in solid tumor patients	Phase 2 NCT00926354
	T-allo10	Stanford University	Donor-derived CD4+ T cells which contain a high proportion of host alloantigen specific Tr1 cells	Therapy to prevent GvHD and induce graft tolerance in hematopoietic stem cell transplant	Phase 1 NCT03198234
	Pegilodecakin (LY3500518 or AM0010)	Eli Lilly and Company	PEGylated recombinant human IL-10	Pancreatic cancer	Phase 3 NCT02923921

Before application of any kind of IL-10 modulation, a very detailed and dynamic assessment of the individual’s immune status must be conducted.

### TGF-β

#### How Does TGF-β Affect Immune Cells?

The TGF-β cytokine exits in three isoforms (β1, β2 and β3) and steers multiple immune processes such as immunosuppression and peripheral homeostasis in autoimmunity and infection (109, 110). Leukocytes mainly express the TGF-β1 isoform (48). It regulates a number of different immune cells and importantly T cells. Their function is mediated by the TGF-β receptor complexes type I and II with TGF-β binding first to type II, then I. Signals of these receptors are mediated by the SMAD transcription factors (43).

Many genetic knockout studies in the past have shown that TGF-β has an important influence on T cells. It can impair T cell proliferation and differentiation, except for Tregs whose proliferation is paradoxically stimulated by TGF-β. It inhibits the proliferation and differentiation of Th1, Th2 and cytotoxic T cells by suppressing, amongst others, the transcription factors T-bet, Gata-3 and c-myc. Further, IL-2 expression as a crucial stimulus for T cell proliferation is suppressed by TGF-β as well (48). TGF-β also promotes the development of Th17, Th9 and T follicular helper cells in conjunction with other Interleukins, but blocks the development of Th1 and Th2 cells (109). The ability of TGF-β to suppress T cell proliferation depends on the activation status of the T cell, which was demonstrated in a study by Cottrez and Groux (111). The proliferation and cytokine secretion in resting, but not in activated T cells is inhibited by TGF-β1. The latter down regulate TGF-β receptor type II, which interestingly could be upregulated again by IL-10 restoring their responsiveness to TGF-β1 (111). TGF-β regulation of T cell survival appears to be dependent on CD28 co-stimulation. In the presence of CD28 agonist TGF-β inhibits T cell apoptosis and enhances their expansion. In its absence, TGF-β inhibits the TCR-stimulated proliferation of naïve T cells (48). Moreover, TGF-β1 supports survival of T cells with memory and effector functions (48), although this seems to contradict its role in the above-mentioned regulation of T cell differentiation.

The role of TGF-β in the regulation of Tregs cells appears to be less contradictory. TGF-β is necessary for the induction of thymic (109) and peripheral Tregs, which develop by antigen-specific stimulation in the presence of TGF-β (112). TGF-β induces the expression of FoxP3 and the production of IL-10 in Tregs (113, 114). Tregs themselves secrete TGF-β forming a positive feedback-loop (50, 115).

Additionally, dendritic cells mediate the induction of peripheral Tregs *via* TGF-β (116). If dendritic cells themselves are exposed to TGF-β in a pro-inflammatory setting, they show impaired production of pro-inflammatory mediators (117). Similar to dendritic cells, TGF-β strongly influences monocytes and macrophages by downregulating their pro-inflammatory functions when activated (48). However, the effect of TGF-β on monocytes and macrophages strongly depends on their activation. In general, TGF-β promotes monocyte function when resting. For example, adhesion molecules, matrix metalloproteinases and IL-6 production is promoted by TGF-β in monocytes (48). In contrast, when activated monocytes become macrophages, TGF-β can have a dampening effect on their pro-inflammatory functions in that it impairs their ability to produce LPS-induced TNF-α, MIP-1α, MIP-2 and reactive oxygen and nitrogen species (48). Another important contribution to uphold inflammation is the ability of macrophages to present antigens to T cells. TGF-β1 downregulates the IFN-γ-induced expression of MHC II mRNA and antigen presentation in macrophages (118). This might serve to prevent excessive and therefore harmful activation of T cells in inflammation.

**Figure 2:**
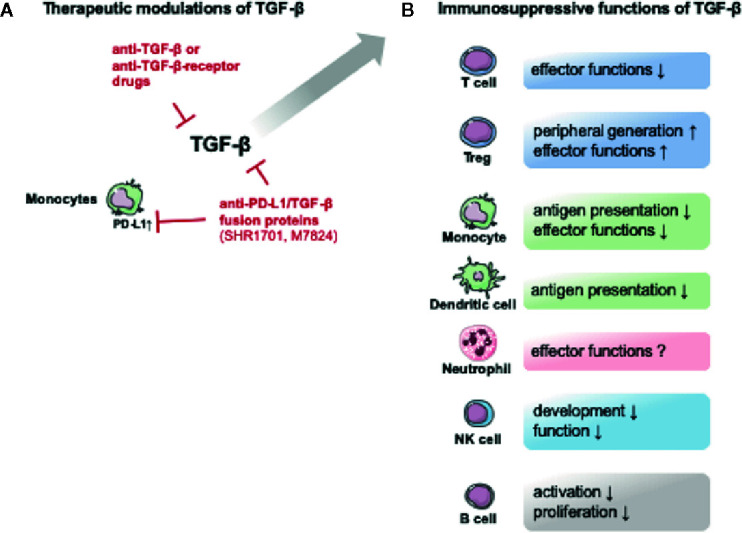
Potential therapeutic TGF-β modulating drugs and the immunosuppressive functions of TGF-β. The figure provides an overview over potential therapeutic approaches and the immunosuppressive effects of TGF-β. **(A)** Several drugs targeting TGF-β or its receptors are currently being tested in non-septic patients. Interestingly an anti- programmed cell death 1 ligand 1 (PD-L1)/TGF-β fusion protein is also being tested. The upregulation of PD-L1 is associated with monocyte dysfunction in post-traumatic and septic immune dysfunction (32). **(B)** TGF-β exerts immunosuppressive effects on both innate and adaptive immune cells. The figure contains adapted graphics from Les Laboratoirs Servier - Medical Art under the terms of the Creative Commons Attribution License (CC BY) for non-commercial use. The use, distribution or reproduction in other forums is permitted: https://creativecommons.org/licenses/by/3.0/legalcode, last accessed July 30th, 2020.

The impact of TGF-β on neutrophils is poorly understood. In cancer, TGF-β polarizes neutrophils from an antitumoral N1 type to a pro-tumoral N2 type (119). However, TGF-β can also promote neutrophil oxidant production (120). Of note, TGF-β is a strong chemoattractant for monocytes, eosinophils, mast cells and neutrophils (48). The immunosuppressive effects of TGF-β on immune cells are outlined in **Figure 2**.

#### Dynamics in Trauma and Sepsis

Given the fact that TGF-β is a potent regulator of immune responses and is considered an important cytokine released by Tregs, the question arises if it is a main regulator in trauma and sepsis and if its dysfunction in these conditions contributes to a disturbed immune response.

Several studies provide contradictory findings. In 2004, Monneret et al. conducted a study examining 38 adult septic patients with septic shock and found that TGF-β levels were decreased compared to normal values and concluded that the general immunosuppressive state they found the patients in is more likely attributed to increased IL-10 levels (121). In addition, the group suggested that no prognostic information could be obtained from TGF-β levels (121). In further support of this, an additional study did not find any prognostic value of TGF-β2 to determine survival in patients with gram-positive septicemia (122). While TGF-β2 levels were increased in the non-survivor group compared to the survivor group in gram-positive septicemic patients, this difference was not statistically significant (122). Gram-negative septicemic patients showed a trend towards decreased TGF-β levels, but this was also not statistically significant (122). Due to the low numbers of patients within each group (11 or more) the study is potentially underpowered to reveal statistically significant differences (122). Of note, TGF-β1 and not TGF-β2 seems to have the largest impact on T cell function and leukocytes mainly express the TGF-β1 isoform (48). A further trial observing gastrointestinal surgery patients with and without infectious complications could not detect serum TGF-β in most of the samples (103). Burn patients examined for IL-10 and TGF-β1 serum levels showed an initial peak in serum TGF-β1 within one day post-burn and a second peak in which the serum levels of the survivors were higher than in the non-survivor group. No differences were found between patients with burned total body surface area (TBSA) of greater or less than 50% (123). However, a limitation in this study of 15 patients is that the surviving group was younger and suffered less severe burn injury with regard to the TBSA (124).

Other studies found elevated TGF-β levels in septic patients. One study has demonstrated elevated TGF-β1 levels in 26 patients at the time of sepsis diagnosis when compared to healthy controls (125). A more recent study from Huang et al. examined Treg function and IL-10 and TGF-β1 serum levels in 106 burned, septic and non-septic patients with a TBSA of more than 30% (45). They revealed a significant increase in TGF-β1 levels in septic patients compared to non-septic patients and in non-survivors compared to survivors (45). They also stratified the patients in three groups depending on the size of the burn. Serum TGF-β1levels were positively associated increasing TBSA (45). From all these studies, the latter examined the biggest population, therefore making it likely that there is indeed a systemic increase in TGF-β in severely injured and septic patients. This view is supported by studies examining specific organ failures in septic populations. A study from De Pablo et al. revealed that increased TGF-β1 levels were positively associated with sepsis induced ARDS with fatal outcome. The TGF-β1 levels were increased in patients 7 days after ICU admission with sepsis and ARDS compared to patients with sepsis without ARDS. In addition, patients with septic shock associated ARDS showed significantly higher TGF-β1 levels in non-survivors than in survivors (126).

In rodent sepsis models, findings concerning TGF-β serum levels are less contradictory then in human studies. Increased levels of TGF-β were found in peripheral blood of septic mice (127). Nullens et al. conducted a murine sepsis model in which lymphocyte depletion occurred in all tissues examined (spleen, mesenteric lymph node, Ileum, colon) at day seven, coinciding with increased levels of IL-10 and TGF-β (128). In a rat sepsis model, increased levels of TGF-β were observed in the circulation and in adherent splenic cells (129). Additionally, supporting the findings in the human study mentioned above from De Pablo et al., Xu et al. reported a protective role of curcumin, most likely by decreasing the expression of TGF-β1 and the SMAD3-dependent signaling pathway (130). Plasma TGF-β1 levels were significantly increased in the septic group and were downregulated by curcumin treatment. Histology showed curcumin treatment prevented some sepsis-induced lung damage (130). These results suggest that TGF-β plays a detrimental role in sepsis most likely by impairing a proper immune response. Yet TFG-β1 might exert detrimental effects on the host by other cells. A study examining the effect of TGF-β on liver cells when challenged with LPS revealed that it promotes the release of proinflammatory cytokines, e.g. IL-6, by the liver cells, which led to a higher mortality (131). Inducible TGF-β1-transgenic mice that express TGF-β1 under control of the C-reactive protein promoter were used in this study (131). Therefore, future studies will be needed to confirm this mechanism in mice that were not genetically modified.

In contrast, other studies found beneficial effects of TGF-β in sepsis. For example, treatment with TGF-β blocked endotoxin-induced hypotension and improved survival in rat models using *Salmonella typhosa* and *Salmonella enteritidis* to induce septic shock and a murine endotoxic shock model (4). Moreover, cardioprotective effects of TGF-β could be shown, as TGF-β reverses the depression of myocyte contraction *in vitro* (132). Another study found TGF-β1 to decrease neutrophil numbers during the onset of LPS-induced acute lung injury (133). This was due to increased apoptosis rather than reduced migration. TGF-β1 did not directly regulate neutrophil apoptosis but instead promoted IL-6 release from mast cells, which promotes neutrophil clearance (133).

Given the partially contradictory findings in the different human studies, there still lacks a definite answer to the question of whether systemic TGF-β release contributes to a systemic detrimental immune disturbance in sepsis. The unambiguous findings in animal models and the findings of Huang et al. including 106 patients, in which TGF-β serum levels were increased in septic patients (45), suggest that TGF-β does play an important role. Even more so as in the current understanding of sepsis, the action of Tregs is considered important for the outcome. Tregs are considered an important source of TGF-β. Moreover, the association of increased TGF-β levels and impaired lung function seems to be relevant in this context, considering that ARDS is a major clinical challenge in septic patients. The local effect of TGF-β in the different tissues has to be examined in the context of trauma and sepsis. This is of importance, as other diseases like rheumatoid arthritis that TGF-β acts very differently when administered locally or systemically (48). Future studies need to address the dynamic expression of this cytokine during sepsis. Several studies mentioned a peak in the beginning of sepsis development (4, 123). This might be due to an overwhelming stimulation of TGF-β producing cells in the beginning of inflammation, or an increase in apoptosis of T cells which are known to create an immunosuppressive environment (134). This could lead to a pathologic immunosuppression at the onset of inflammation. Lastly, the role of TGF-β may differ in sepsis and sterile trauma. Therefore, future studies should also address differences in non-sterile and sterile settings to evaluate appropriate therapeutic action in the different diseases.

#### Effects and Drivers of Pathological Release in Trauma and Sepsis

Assuming an adequate response to an infection, we postulate the role of TGF-β as follows: First, it acts as a chemoattractant for myeloid cells in the very beginning of infection to promote initial innate immune response. This underlying premise is based upon that TGF-β acts in a stimulatory manner on resting monocytes (48). Further, TGF-β impairs the activation of naïve T cells but can be overridden by CD28 co-stimulation in addition to low IL-2 doses (48). The team around Li et al. argues that this circumstance serves as a threshold to divide between self-antigen driven steady state inflammation, which is hindered by TGF-β, and a pathological condition in which the above mentioned stimuli override TGF-β inhibition to guarantee a proper immune response to the infection (48). As stated above, TGF-β curbs macrophages in inflammation which might serve to restore hemostasis and prevent excessive inflammation.

The data suggest that these regulated dynamics are severely impaired in sepsis. In the study from Hiraki et al., a murine abdominal sepsis model was utilized with a TGF-β depleting antibody administered 6 h after the intervention, which lead to improved survival of the mice (103). The group also showed a positive correlation between the percentage of Tregs of all CD4 T cells and the serum TGF-β levels (103). It is known from human clinical studies that an increased percentage of Tregs is correlated with worse outcome (45, 135). The group around Hiraki et al. therefore postulates that the disruption of TGF-β leads to a decrease of Treg abundance which might be harmful in the development of sepsis (103). It is also possible that TGF-β itself plays a detrimental role by depressing T cell functions and disrupting the positive feedback loop that promotes Treg proliferation (50, 115). The study did not examine which cell type provides the major source of TGF-β. It is assumed that the initial release of auto- and or alloantigens released when infection occurs leads to such a massive TGF-β release that homeostasis as described above can no longer be restored. Currently, it is not clear whether this is driven by DAMPs or PAMPs. In the study, the administration of the depleting antibody led to the restoration of this homeostasis. Of note is that a single administration was enough. A current study from Zeng et al. supports these findings (136). An anti-TGF-β antibody was administered, which led to increased IL-2 and IFN-γ levels. This was considered beneficial for the outcome. The authors, however, neglected to provide the timepoint of the anti-TGF-β-antibody administration (136).

Altogether, these studies contribute to the understanding of the role of TGF-β and Tregs in the initial phase of sepsis. However, it is not possible to draw a definite conclusion from what is currently known, given the fact that the effects of TGF-β differ a lot depending on location, timing and the activation status of the cells it affects as described in the section above.

#### Potential Therapeutic Modulation of TGF-β

Currently, TGF-β dynamics in human sepsis studies need to be expanded before a therapeutic modulation of TGF-β or its receptors should be applied. The focus of studies should be on the dynamics and the origin of sepsis (organ/tissue). In current clinical trials, a number of drugs are tested that might prove useful in the future. An overview of current TGF-β modulating drugs is provided in **Table 2**. These include direct anti-TGF-β antibodies (e.g. Fresolimumab: Human monoclonal anti-TGFβ-antibody (Non-small cell lung carcinoma), Phase 1/2 NCT02581787) and receptor blockage (Galunisertib (LY2157299): small molecule inhibitor of the kinase domain of Type 1 TGFβ receptor (solid tumor, non-small cell lung cancer, hepatocellular carcinoma), Phase 1/2 NCT02423343). Interestingly, an anti- programmed cell death 1 ligand 1 (PD-L1)/TGF-β fusion protein is also being tested (e.g. SHR-1701: anti-PD-L1/TGFβ fusion protein (advanced solid tumors), Phase 1 NCT04324814). If TGF-β blockage proves beneficial in trauma or sepsis, application of this drug would be a very interesting approach as the upregulation of PD-L1 is associated with monocyte dysfunction and upregulation of its ligand programmed cell death protein 1 (PD-1) in lymphoid cells is a detrimental hallmark of post-traumatic and septic immune dysfunction (32). A first anti-PD-1 antibody (nivolumab) has already been tested for safety in septic patients (Phase 1b NCT02960854) (137). The potential therapeutic modulations of TGF-β are outlined in **Figure 2**.

**Table 2:** Clinical Trials targeting TGF-ß or its receptor.

Cytokine	Drug	Sponsor	Description	Indication	Clinical trial
**TGF-β**	Juvista (Avotermin)	Renovo	TGF-β3	Wound healing	Phase 2 NCT00629811
	TGFβ-resistant LMP-specific cytotoxic T lymphocytes	Baylor College of Medicine	TGFβ-resistant LMP-specific cytotoxic T lymphocytes	Lymphoma	Phase 1 NCT00368082
	LY3200882	The Netherlands Cancer Institute	TGFβRI small molecule inhibitor	Colorectal metastatic cancer	Phase 1/2 NCT04031872
	TGFβ-resistant HER2/EBV-CTLs	Baylor College of Medicine	TGFβ-resistant HER2/EBV-Cytotoxic T cells	Treatment of Her2 positive malignancy	Phase 1 NCT00889954
	AVID200	Formation Biologics	TGFβ-receptor ectodomain-IgG Fc fusion protein inhibitor of TGFβ	Malignant solid tumor	Phase 1 NCT03834662
	TGFβ2 Antisense-GMCSF Gene Modified Autologous Tumor Cell (TAG) Vaccine	Mary Crowley Medical Research Center	TGFβ2 Antisense-GMCSF Gene Modified Autologous Tumor Cell (TAG) Vaccine	Advanced metastatic carcinoma	Phase 1 NCT00684294
	AP 12009	Isarna Therapeutics GmbH	Phosphorothioate antisense oligodeoxynucleotide specific for the mRNA of human TGFβ2	Pancreatic neoplasms, melanoma, colorectal neoplasms	Phase 1 NCT00844064
	GPC3 and/or TGFβ targeting CAR-T cells	Second Affiliated Hospital of Guangzhou Medical University	Third/fourth generation of CAR-T cells that target GPC3 (GPC3-CART cell) and/or soluble TGFβ (GPC3/TGFβ-CART)	Hepatocellular carcinoma, lung cancer	Phase 1 NCT03198546
	P144 cream	ISDIN	TGFβ1-inhibitor, designed to block interaction between TGFβ1 and TGFβ1 type III receptor	Healthy (tolerability and bioavailability)	Phase 1 NCT00656825
	GC 1008	National Cancer Institute (NCI)	Human anti-TGFβ- monoclonal antibody	Renal cell carcinoma	Phase 1 NCT00923169
	M7824	National Cancer Institute (NCI)	Bifunctional anti-PD-L1/TGFβ Trap fusion protein	Head and neck cancer	Phase 1/2 NCT04247282
	Fresolimumab	Maximilian Diehn, Stanford University	Human monoclonal anti-TGFβ-antibody	Non-small cell lung carcinoma	Phase 1/2 NCT02581787
	Galunisertib (LY2157299)	Eli Lilly and Company	Small molecule inhibitor of the kinase domain of Type 1 TGFβ receptor	Solid tumor, non-small cell lung cancer, hepatocellular carcinoma	Phase 1/2 NCT02423343
	Vactosertib (TEW-7197)	MedPacto, Inc.	Inhibitor of the serine/threonine kinase of the TGFBR1	Recurrent advanced urothelial carcinoma	Phase 2 NCT04064190
	GS-1423	Gilead Sciences	Anti-CD73- TGFβ-Trap bifunctional antibody	Advanced solid tumors	Phase 1 NCT03954704
	PF-03446962	Pfizer	Monoclonal antibody against Activin Receptor-Like Kinase-1 (ALK1), a type I subclass of the TGFβ receptor	Advanced solid tumors	Phase 1 NCT00557856
	SHR-1701	Atridia Pty Ltd.	Anti-PD-L1/TGFβ fusion protein	Advanced solid tumor	Phase 1 NCT04324814
	SRK-181	Scholar Rock, Inc.	Inhibitor of the activation of latent TGFβ1	Locally advanced or metastatic solid tumors	Phase 1 NCT04291079
	NIS793	Novartis Pharmaceuticals	Anti-TGFβ-antibody	Breast-, lung-, hepatocellular-, colorectal-, pancreatic-, renal-cancer	Phase 1 NCT02947165

### TSLP

#### How Does TSLP Affect Immune Cells?

Thymic stromal lymphopoietin (TSLP) is a member of the IL-2 cytokine family and is closely related to IL-7 (138). It appears in two variants, a long form of TSLP (lfTSLP) and a short form (sfTSLP). These two forms do not derive from alternative splicing but are promoted by different putative promoter regions (139). Their effects on immune cells differ widely. Of note, many articles do not differentiate between the long and the short form of TSLP (139). In a review of Bjerkan et al., the authors summarized that the in absence of infection the short form is steadily expressed and the long form is absent (139). The short form seems to provide homeostasis especially on healthy barrier tissue like in the skin and gut. However inflammation can induce the expression of the long form and the short form becomes downregulated (139).

The diseases TSLP are best studied in are asthma, inflammatory bowel diseases and cancer, in which a Th2 dominated inflammation occurs or Th2 cells promote cancer cell survival (138). Keratinocytes, epithelial cells, mast cells, smooth muscle cells, fibroblasts and dendritic cells are producers of TSLP (140). However, it can also be produced by mast cells, human monocytes, macrophages and granulocytes, murine basophils and cancer cells (141). Unlike cells producing TSLP, which are comparably few, many more cells become activated by TSLP. Cells known to become activated by TSLP, are dendritic cells, innate lymphoid cells 2 (ILC2), CD4 T and Th2 cells, natural killer T cells, CD8 T cells and B cells, regulatory T cells, eosinophils, neutrophils, murine (but not human) basophils, monocytes, mast cells, macrophages, platelets, and sensory neurons (141).

Mouse and human TSLP bind to the combination of the IL-7 receptor α chain (IL-7Rα) and the TSLPR (TSLP receptor) chain, but only rarely to TSLPR alone (142). An extensive review about the intracellular pathways of TSLPR signaling would exceed the scope of this review, but was compiled by Zhong et al. and Yu et al. (143, 144). The lfTSLP receptor is expressed on several immune cells such as dendritic cells, T cells, B cells, natural killer cells, monocytes, basophils, eosinophils, and epithelial cells (139). The TSLPR was also found on tissue in heart, skeletal muscle, kidney, and liver (142). On CD4 T cells the TSLPR is upregulated upon TCR stimulation assuming a regulative role in inflammation (142). It is believed to portray an endothelial reaction to endogenous and exogenic triggers and drive a Th2-dominated inflammatory reaction mediated by dendritic cells. However, these Th2 cells also produce TNF-α, but not IL-10 (145).

It has been demonstrated that TSLP can exert potent antimicrobial effects. It could be shown that TSLP inhibits the growth of *Escherichia coli, Pseudomonas aeruginosa, Staphylococcus aureus, Staphylococcus epidermidis* and fungal species (139). Furthermore, TSLP augments ROS production in neutrophils by triggering the complement C5 system which enhances killing of *S. aureus* in human and mice (146).

#### Dynamics in Trauma and Sepsis

**Figure 3:**
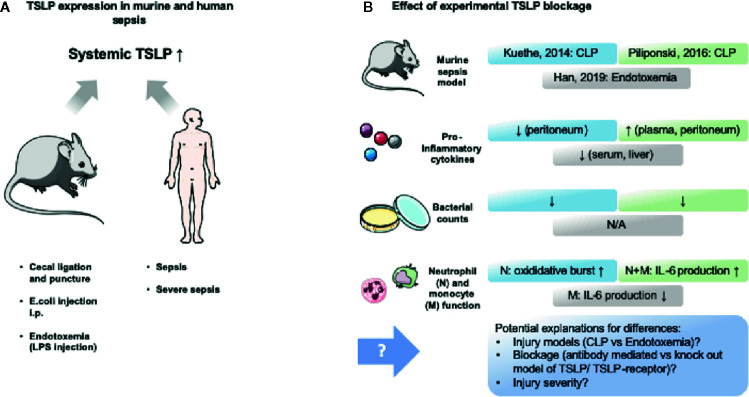
TSLP expression in murine and human sepsis and its experimental modification. Murine and human sepsis acutely lead to increase of systemic TSLP blood levels. Murine studies blocking TSLP provided contradictive results, which might be explained by differing methodology. **(A)** Several murine sepsis (cecal ligation and puncture and intraperitoneal injection of live bacteria) and endotoxemia (intraperitoneal injection of lipopolysaccharide (LPS)) models, as well as human observational trials in sepsis and severe sepsis revealed that TSLP levels are elevated in the blood within 24 h (44, 147, 148). **(B)** Three murine studies (44, 147, 148) assessed the effect of TSLP blockage and provided contradictive results that are highlighted in this figure. An explanation for these differences might be found in the different methods that were used. The figure contains adapted graphics from Les Laboratoirs Servier - Medical Art under the terms of the Creative Commons Attribution License (CC BY) for non-commercial use. The use, distribution or reproduction in other forums is permitted: https://creativecommons.org/licenses/by/3.0/legalcode, last accessed July 30th, 2020.

TSLP has not been studied extensively in sepsis to date. Several studies provide conflicting results concerning the effect of TSLP in animal sepsis models or human studies (44, 47, 147, 148). These findings are summarized in **Table 3** and visualized in **Figure 3**. Undisputed findings of the mentioned murine and human studies are that TSLP serum levels increase within the initial phase of the inflammation. Depending on the injury model or clinical circumstances, this initial peak ranges from 6 (147) to 24 h (44) and seems to decrease with the course of the inflammation.

**Table 3:** The effect of TSLP in sepsis.

Sepsis Model/Intervention	Intervention/Mouse model	Murine or human	Mortality	Cytokine expression	Other parameters	Study
CLP	Anti TSLP-Ab	M	↓	TNF-a, IL-17 (ip) ↓	Neutrophil no (ip) ↑ Neutrophil ox. burst ↑ CFU ↓	Kuethe et al. (147)
CLP	Anti TSLP-Ab	M			Drop in body temp ↑ CFU ↔	Piliponsky et al. (44)
CLP	TSLP administration	M		TNF, IL-17A, IL-6, KC (plasma and ip) ↓	Drop in body temp ↓ Neutrophil no (ip) ↓ CFU (ip) ↔ (blood) ↑	
CLP	TLSPR-/-	M	↑	TNF, IL-17A, IL-6, KC (plasma and ip) ↑	Drop in body temp ↑ Neutrophil/Macrophage no (ip) ↑ CFU (ip and blood) ↓	
CLP	Lys-Cre^+^; Tslpr ^fl/fl^ (reduced TSLPR expression)	M	↑	TNF, IL-17A, IL-6, KC (plasma) ↑	Neutrophil/Macrophage no (ip) ↑ CFU (ip and blood) ↓ IL-6 (within Neutrophils and Macrophages) ↑	
LPS stim (*in vitro*: Neutrophils)	TSLP administration	H			IL-6 (within Neutrophils) ↓	
Sepsis	LPS stim (*in vitro*)	H			IL-6, TNF-α, IL-1b (non-classical and intermediate Monocytes) (in patients above av. TSLP serum levels vs lower TSLP Serum levels) ↑	Yu et al. (47)
Sepsis	PMA (*in vitro*)	H			Neutrophil ox burst and phagocytosis ↔ (in patients above av. TSLP serum levels vs lower TSLP Serum levels) ↑	
Sepsis	Cell stim (*in vitro*)	H			TNF-a, IFN-y (CD4 T cells) (in patients above av. TSLP serum levels vs lower TSLP Serum levels) ↑	
LPS injection (ip)	TSLP siRNA injection (reduces TSLP levels) Or TSLP-/-	M		IL-6, VEGF, ICAM-1, and MIP2, TNF-a (serum, liver)↓		Han et al. (148)
LPS injection (ip)	TSLP administration	M		IL-6 (serum) ↑		
LPS stim (*in vitro*: Monocytes (RAW264.7, WT and TSLP-/-))	TSLP siRNA transfection: RAW264.7 cells (reduces TSLP levels). WT: Anti-TSLP-Ab TSLP-/-: no intervention	M			IL-6, ICAM-1, MIP-2, TNF-a, NO production in all types on Monocytes ↓	
						
CLP	–	M		TSLP (serum) ↑		Kuethe et al. (147)
CLP	–	M		TSLP (plasma, ip) ↑	Neutrophil TSLPR expression ↑	Piliponsky et al. (44)
Severe Sepsis	–	H		TSLP (plasma) ↑		
Sepsis	–	H		TSLP (serum) ↑ (in patients with hyperleukocytosis (HL) and high neutrophil ratio (HNR) vs without HL and HNR)	Mortality ↑ (in patients above average TSLP serum levels vs lower TSLP serum levels)	Yu et al. (47)
Sepsis		H		TSLP (serum) ↑		Han et al. (148)
LPS, E.coli injection (ip)		M		TSLP (plasma, ip) ↑		

Up to now, none of the studies included a long-term analysis of TSLP levels and none of the studies conducted in sepsis differentiated between the long and the short TSLP form. As the long form is considered to become upregulated in inflammatory settings (139), it is likely that the long form is what was found to be elevated in the serum and peritoneum. This is of note as it might provide an explanation for the conflicting results found in the TSLP/sepsis studies concerning inflammatory and immunosuppressive actions of TSLP.

The first study that provided an examination of the effect of TSLP in sepsis was performed by Kuethe et al. (147). This group found that TSLP blockade decreased mortality and dampened the production of inflammatory cytokines (147). A similar study was conducted by Piliponsky et al. in 2016 (44). In contrast to the study of Kuethe et al. the administration of anti-TSLP-Ab in a murine abdominal sepsis model lead to an increase in morbidity and did not improve bacterial clearance (44). The authors argue that this may be explained by different injection times and inflammation dynamics. Otherwise, they confirmed the immunosuppressive role of the TSLP-TSLPR interaction as TSLPR-/- mice displayed increased intraperitoneal levels of TNF-a, IL-6, IL-17A and KC after onset of abdominal sepsis (44). However, TSLPR-/- mice had impaired bacterial clearance leading the authors to compare the effect of TSLPR with the effect that can be seen in IL-10 deficient mice succumbing to sepsis due to impaired bacterial clearance (44). Piliponsky et al. depicted that TSLP mainly dampens myeloid cytokine production, which then contributes to reduced morbidity by limiting inflammation (44). Overall, they conclude that the upregulation of TSLP in sepsis aims to restore immune hemostasis by dampening inflammation (44). In contrast, Yu et al. demonstrated a negative correlation between serum TSLP levels in patients with high ratio of neutrophils and increased mortality in a septic patient cohort (47). Moreover, they showed TSLP induces inflammation in neutrophils and T lymphocytes and certain monocyte subtypes (intermediate and non-classical) leading to increased production of e.g. IL-6, TNF-α and IFN-γ (47). They therefore conclude that TSLP has a pro-inflammatory effect on monocytes and lymphocytes but does not improve antibacterial clearance of neutrophils, as phagocytosis and respiratory burst were unchanged (47). In line with these findings is a study by Han et al. They revealed that TSLP-deficient mice show lower levels of IL-6, VEGF, ICAM-1, and MIP2 in serum in LPS challenged mice (148). They also demonstrated TSLP upregulates macrophage-mediated inflammation as TSLP neutralization and TSLP siRNA silencing reduced the production of IL-6, TNF-α and NO after LPS stimulation. Moreover, they showed LPS or *E. coli* stimulated macrophages *via* TLR4 produce TSLP (148). The finding of decreased proinflammatory cytokines in TSLP-deficient mice contradicts the findings of Piliponsky et al. and Kuethe et al., although Han et al. in part used the same experimental approach (Anti-TSLP-Ab) (44, 147, 148). The team of Han et al. argues that the different septic models (LPS vs cecal ligation and puncture (CLP)) may provide an explanation for this. They summarize that TSLP triggers proinflammatory reactions especially of macrophages and might lead to organ dysfunction in sepsis (148).

Taken together, these studies provide surprisingly conflicting data about the effect of TSLP in sepsis. It will be critical to consider the differences between the models to explain these results. The CLP model conducted by Kuethe et al. and Piliponsky et al. constitutes a physiologically more relevant model as it includes pathogen related tissue damage, phagocytic uptake of pathogens by neutrophils and macrophages, as well as the release of other pathogenic antigens compared to the administration of LPS alone, which lacks all these important characteristics. Moreover, Han et al. used relatively low LPS doses (148) which may not induce an inflammatory reaction as strong as in the CLP model. This may indicate a different role of TSLP depending on the injury severity and inflammatory milieu. However, until further direct experimental comparisons, this remains speculative.

The following points should be addressed in further studies to clarify the role of TSLP in sepsis. First, a discrimination between the short and the long form should be conducted in future studies as their effects are considered to differ (139). Secondly, studies should differentiate between the severity of sepsis, as Piliponsky as well as Yu et al. reported differences in the effect of TSLP regarding the severity of the disease (44, 47). Thirdly, none of the studies so far address the release of TSLP by endothelial cells, which are considered the main producers (140). The effect of TSLP on macrophages and endothelial cells seem most important as they might steer potential pro- or anti-inflammatory effects. Lastly, the dynamics of the septic disease should be examined, as this may also be a potential cause of the current conflicts in the data.

#### Effects and Drivers of Pathological Release in Trauma and Sepsis

The production of TSLP is triggered by allergens, pro-inflammatory cytokines, viruses, bacteria, fungi and tryptases (141). With respect to a septic setting, the challenge of epithelial cells with bacteria is likely the most relevant. This poses the important question of whether the stimulation occurs directly by microbes or in an indirect manner mediated by consecutively upregulated pro-inflammatory cytokines. In a study performed by Lan et al. it is demonstrated that *Staphylococcus aureus* directly induces epithelial cell-derived TSLP release *via* TLR-2-binding (149). Although not in a septic model, this might explain the upregulation of TSLP in sepsis (149). If TSLP is considered to restore immune hemostasis in sepsis, which still has to be proven due to the conflicting results past studies provided, then overwhelming microbial challenge might lead to detrimentally high release of TSLP. This could provide an explanation for conflicting data as lower levels might restore homeostasis, but by surpassing a certain threshold, TSLP may trigger inflammation and therefore worsen outcome. However, this currently remains a matter of speculation.

#### Potential Therapeutic Modulation of TSLP

As discussed in the previous section more research is needed to characterize the effect of TSLP in trauma or sepsis. However, antibodies directed against TSLP are currently tested for safety in clinical trials aiming to treat allergic diseases (e.g. MEDI9929: human monoclonal antibody immunoglobulin IgG2λ directed against TSLP (asthma), Phase 2 NCT02698501). An overview of current TSLP modulating drugs is provided in **Table 4**. These might prove useful in clinical trials when dynamics and functions of TSLP in trauma and sepsis have been elucidated.

**Table 4:** Clinical Trials targeting TSLP.

Cytokine	Drug	Sponsor	Description	Indication	Clinical trial
**TSLP**	MEDI9929	Celeste Porsbjerg	Human monoclonal antibody immunoglobulin IgG2λ directed against TSLP	Asthma	Phase 2 NCT02698501
	AMG 157 (MEDI9929)	National Institute of Allergy and Infectious Diseases (NIAID)	Anti-TSLP antibody	Cat Allergy	Phase 1/2 NCT02237196

## Conclusion

Sepsis is responsible for one out of three in-hospital deaths (150) while trauma is still the leading course of death for people under the age of 46 in the United States (151). The following discusses the two most pressing challenges for these pathophysiologies:

1) The development of methods to stratify patients, to evaluate which of three clinical trajectories the respective patient will follow: early death, rapid recovery or chronic critical illness (19). Promising strategies are phenotyping innate and adaptive immune cells, using flow cytometry (32), assessing genetic or transcriptional changes within innate and adaptive immune cells (152), evaluating cytokine levels and their ratio’s (31) and most importantly use functional assessment of immune cell behavior (46, 153). The use of functional assays has proven useful to examine the capability of cells to produce cytokines in response to stimuli and thus enables the assessment of the current immune status (46). Moreover, patient endotypes within the sepsis and trauma populations should be defined (10). These endotypes should be studied to determine if they are associated with good or bad clinical outcomes. Knowing the endotype associated with the immune status should allow for a more personalized and effective therapeutic strategy. All of the clinical, phenotypic and functional assays should be analyzed in combination with the standard clinical data on the ICU’s. We believe that dynamic immune monitoring prospectively should become a standard in intensive care medicine.

2) If proper stratification of patients is successful the next step would be to therapeutically address immune perturbation. Currently the use of three cytokines is being tested to improve immune function in sepsis and infection: IFN-γ to treat sepsis-induced immunoparalysis (Phase 3, NCT01649921), granulocyte-macrophage colony-stimulating factor (GM-CSF) to decrease ICU acquired infections (Phase 3, NCT02361528), IL-7 to restore lymphocyte counts in sepsis patients (Phase 2, NCT02640807). Beside cytokine modulation the blockage of the programmed cell death protein 1 (PD-1), a checkpoint inhibitor with elevated expressed in sepsis patients (154), might prove beneficial, regarding its ability to hinder T cell apoptosis in murine sepsis model and *in vitro* human sepsis studies (155–157). Moreover, the blockage of cytotoxic T-lymphocyte-associated protein 4 (CTLA-4), another immune checkpoint receptor overexpressed in sepsis patients (155, 158) that improved T cell survival in mice (159), might provide a therapeutic approach.

Other than the above mentioned, there are no current approaches to clinically test modulation of immunosuppressive cytokines, such as IL-10, TGF-β and TSLP in sepsis or trauma. Immunosuppression is responsible for significant numbers of deaths in sepsis and trauma patient populations (13). It is therefore key to further examine how immunosuppressive cytokines affect immune hemostasis and how they can be beneficially modulated.

As highlighted in this review the effect of the potent anti-inflammatory cytokines IL-10, TGF-β, and TSLP depends on dynamics, dose and receptive cell type. Thus, the following prerequisites for these specific cytokines should be met to approach pharmaceutical therapeutic modulation of IL-10, TGF-β, and TSLP: 1) Future pre-clinical or clinical studies should focus on temporal expression of these cytokines. 2) The collective literature shows improved predictive power when a combination of cytokines was assessed to predict outcome (31, 160, 161). Therefore, they should be evaluated relatively to each other and other pro-inflammatory cytokines. 3) Future research should focus on which cells are the main contributors to the systemic release and functionally assess which stimuli drive the release of the respective cytokine, so the specific cell function can be therapeutically modulated.

If these criteria are met and a clear characterization of cytokine behavior emerges, we suggest translating results and immune modulating therapeutics from other pathophysiologies, such as autoimmune diseases and cancer, to sepsis and trauma. These promising treatments that are being tested in these diseases potentially share common mechanisms. Thus, these FDA approved drugs for cancer and autoimmunity could potentially be trialed in the setting of sepsis or trauma to address pathological cytokine release of IL-10, TGF-β, and TSLP leading to disbalanced immunosuppression.

## Data Availability Statement

The original contributions presented in the study are included in the article/supplementary material. Further inquiries can be directed to the corresponding author.

## Author Contributions

CB and CC outlined the review. CB drafted the review, tables, and figures. CB and NB designed the figures. CC, NB, CS, MH, and PC reviewed the drafted review. The latter three especially providing critical clinical input concerning therapeutic approaches. All authors contributed to the article and approved the submitted version.

## Funding 

This work was supported by funding from the Deutsche Forschungsgemeinschaft (German Research Foundation) (BE 7016/1-1) (CB).

## Conflict of Interest

The authors declare that the research was conducted in the absence of any commercial or financial relationships that could be construed as a potential conflict of interest.
